# Effects of reduced-protein diets with protease supplementation on growth, carcass yield, intestinal morphology, organ development, nutrient digestibility, and blood biochemical of broiler chickens

**DOI:** 10.1093/tas/txad098

**Published:** 2023-09-05

**Authors:** Xuerong Song, Muhsin Al Anas, Asih Kurniawati, Chusnul Hanim, Muhlisin, Muhammad Anang Aprianto, Abd. Majid Ahmad Madani, Qijun Wang, Hao Chen

**Affiliations:** Wuhan Sunhy Biology Co., Ltd., Wuhan, P.R. China; Department of Animal Nutrition and Feed Science, Faculty of Animal Science, Universitas Gadjah Mada, Yogyakarta 55281, Indonesia; Department of Animal Nutrition and Feed Science, Faculty of Animal Science, Universitas Gadjah Mada, Yogyakarta 55281, Indonesia; Department of Animal Nutrition and Feed Science, Faculty of Animal Science, Universitas Gadjah Mada, Yogyakarta 55281, Indonesia; Department of Animal Nutrition and Feed Science, Faculty of Animal Science, Universitas Gadjah Mada, Yogyakarta 55281, Indonesia; Department of Animal Nutrition and Feed Science, Faculty of Animal Science, Universitas Gadjah Mada, Yogyakarta 55281, Indonesia; Department of Animal Nutrition and Feed Science, Faculty of Animal Science, Universitas Gadjah Mada, Yogyakarta 55281, Indonesia; Wuhan Sunhy Biology Co., Ltd., Wuhan, P.R. China; Wuhan Sunhy Biology Co., Ltd., Wuhan, P.R. China

**Keywords:** broilers, growth performance, low-protein diet, nutrient digestibility, protease

## Abstract

This study was conducted to evaluate growth performance, carcass yield, intestinal morphology, organ development, nutrient digestibility, and blood biochemical parameters of broiler fed 1% reduced-protein diets with/without protease supplementation. A total of 1,120 one-day-old male broiler chickens with average initial body weight (**BW**), 46.45 ± 0.49 g, were divided into five groups with seven replications and 32 birds per replication. The treatment varied according to the protein and protease enzyme levels: positive control (**PC**), negative control (**NC**, PC with reduction of 1% protein), PC supplemented with 50 g/t protease (PC + 50), NC supplemented with 50 g/t protease (NC + 50), and NC supplemented with 100 g/t protease (NC + 100). The results showed that there was no significant effect of 1% reduced-protein diets, with or without protease on feed intake, final BW, average daily gain, feed conversion ratio, and nutrient digestibility. The changes in dietary protein level and supplementation of protease did not affect carcass yield, but significantly affected abdominal fat content, PC + 50 group had significantly lower abdominal fat content than NC-based diet including NC, NC + 50, NC + 100. Reduced-protein with protease supplementation strongly affected organ weight, especially on day 21: the pancreas was heavier in PC and NC + 50 group than other groups, spleen was heaver in NC group than in NC + 100 group, thymus was heavier in NC + 50 group than in PC, NC and NC + 100 group, small intestine was heavier in NC + 50 and NC + 100 group than in PC group, and large intestine was also heavier in NC + 50 group than in NC group. Villus height sampled at 35-d was significantly increased with protease supplement, and which was significantly higher in NC + 100 group than NC group. Regarding on blood metabolites, only urea and uric acid were affected by the reduction of dietary protein, broiler fed PC diet had higher urea and uric acid content than fed NC diet. In conclusion, supplementation of 50 g/t protease in 1% reduced-protein diets does not negatively affect on growth, nutrient digestibility, carcass yield, organ development, and blood metabolites. Moreover, supplementation of protease in low-protein diet could effectively promote organ development and benefit intestine morphology.

## Introduction

Feed costs account for more than 70% of total production costs in the poultry industry, where the cost of production is determined by the price of ingredients and the effectiveness with which they are used (Yaqoob et al., 2022). Protein is the second-most substantial nutrient for poultry formulation as a supply of amino acids and the most expensive nutrient for the poultry diet. The feed crude protein (**CP**) content exceeding optimal levels is considered uneconomical and environmentally unfriendly. As digestion and absorption of nutrients decrease, pathogens can use the excess protein in the lower digestive tract as a food source, thereby increasing the risk of disease. Moreover, excess protein in the body will be broken down and used for energy or excreted as uric acid (Qiu et al., 2023). Soybean meal is a major source of protein in poultry feed formulation because it is high in protein content and complete in amino acids, making the poultry sector highly dependent on it (Park et al., 2020). Recently, there has been a dramatic increase in the price of soybean meal because of unpredictable supply and production disruptions due to climate change and trade frictions between the countries (Shad et al., 2022). Reducing CP levels in animals’ feed formulation is regarded as one of the most effective recommendations for reducing production costs and environmental load (Kobayashi et al., 2013). However, the availability of amino acids mainly essential amino acids becomes insufficient (Jabbar et al., 2021a) thus causing slow growth and less efficient use of feed (Bregendahl et al., 2002). Recently, many reports have shown that a low-protein diet supplemented with amino acids could improve protein utilization in broiler production without any negative impacts on productivity, carcass yield, breast muscle, and reducing nitrogen excretion, which seems promising for constructing a low-protein diet and thus easing the cost pressures of animal farming (Amer et al., 2021).

Another effective way to improve dietary protein utilization in commercial feed is by the inclusion of exogenous protease enzyme, whose effects have been verified experimentally on increasing protein digestibility and then enhancing protein use (Park et al., 2020; Jabbar et al., 2021b; Shad et al., 2022; Vieira et al., 2023). Endogenous protein enzymes are secreted by the animal themselves and their concentration are not sufficient for efficient protein digestion, especially in young animal or animal under stress. The undeveloped intestine of the young animal causes the limited secretion of digestive enzymes, which causes the incomplete digestion of dietary protein and other nutrients (Nitsan et al., 1991; Noy and Sklan, 1995; Barua et al., 2021a; 2021b). Now, for industrial feeding, the grower and finisher also face digestive problems with overloading feed intake (Jabbar et al., 2021a). This is the reason exogenous protease is widely used in poultry, swine, and aquatic feed (Cowieson and Roos, 2013; Lee et al., 2018; Feng et al., 2023). Many studies have validated that exogenous proteases could increase protein hydrolysis and decrease antinutritional factors such as lectins or trypsin inhibitors, eventually reducing the need for amino acids and energy from diet (Doskovic̈ et al., 2013). Supplementation of protease can improve intestinal integrity and protein digestibility as a result enhance growth and production performance (Cowieson et al., 2018), which can also reduce environmental impacts and maximize the use of protein sources (Leinonen and Williams, 2015). However, in other research, supplementation of protease has side effects on animals’ growth performance (Walk et al., 2018) through inhibiting the secretion of endogenous protease (Inborr, 1990; Mahagna et al., 1995) and causing damage to the integrity of the intestinal tissue structure (Spaendonk et al., 2017). The facts demonstrated that the effectiveness of exogenous proteases is highly related to its inclusion levels. There is currently a lack of understanding regarding the dose-dependent relationship of commercial proteases for animal growth, organ weight, intestinal health, and nutrient digestibility. Thus, the aim of the present study is to determine the effect of reduced-protein diets with different protease levels on broiler performance, carcass yield, abdominal fat, organ weight, blood biochemical, intestinal morphology, and nutrient digestibility. We hope to provide data support for the accurate application of protease, and contribute to sparing valuable protein sources and formulating low-protein diet for broilers.

## Materials and Methods

### Ethics Statement

All animal experiment protocols were approved by the Research Ethics Commission, Faculty of Veterinary Medicine, Universitas Gadjah Mada, Indonesia.

### Animal, Diets, and Experimental Design

A total number of 1,120 one-day-old male chicks of the New Lohmann Indian River (MB 202) with an average initial body weight 46.45 ± 0.49 g were used in the present study. The birds were divided into five dietary treatments in a randomized complete design. There were 1) positive control (**PC**, corn–soybean meal-based diet), 2) negative control (**NC**, PC with reduction of 1% dietary protein), 3) PC + 50 g/t protease (PC + 50), 4) NC + 50 g/t protease (NC + 50), and 5) NC + 100 g/t protease (NC + 100). Sunpro is a commercial protease product, from Wuhan Sunhy Biology Co., Ltd., Wuhan, China, which was used as the protease source in the present study. Each treatment group was divided into seven replications, with 32 birds in each replicate pen. In this experiment, the basic diet was corn–soybean meal diet is shown in Table 1. The feeds were processed by the Buhler machine, using conditioning temperature between 80 and 85 °C. The feed and drinking water were provided ad libitum for a 5-wk rearing period. The starter diets were given on days 0 to 10, while the grower and finisher diets were given on days 11 to 21 and 22 to 35, respectively. The composition of the experimental diets is shown in Table 1. Room lighting, relative humidity, and temperature based on the closed house system that followed the recommendations of the Indian River broiler management handbook Aviagen (2018). The room was maintained at 30 °C for 3 d and then reduced to 2.5 °C/wk until reached 20 °C. In the early stage of growth up to 7 d, lighting programs provide for a long day with a lengthy day consisting of 23:1 (L:D) h. After 7 d, around 5 h of darkness may be optimal (4 to 6 h).

**Table 1.** Ingredients and nutrients composition of experimental diets

Items	Starter					Grower					Finisher				
	PC	NC	PC + 50	NC + 50	NC + 100	PC	NC	PC + 50	NC + 50	NC + 100	PC	NC	PC + 50	NC + 50	NC + 100
Ingredients, %															
Corn	55.59	57.35	55.59	57.35	57.35	59.59	61.68	59.59	61.68	61.68	62.82	64.52	62.82	64.52	64.52
Soybean meal	31.18	29.04	31.18	29.04	29.04	25.87	23.32	25.86	23.32	23.31	16.69	14.46	16.68	14.45	14.45
Corn gluten meal	2.30	2.40	2.30	2.40	2.40	1.80	2.00	1.80	2.00	2.00	1.27	2.00	1.27	2.00	2.00
Meat bone meal	2.10	2.10	2.10	2.10	2.10	2.40	2.68	2.40	2.68	2.69	4.31	4.42	4.31	4.42	4.42
Bran Pollard						1.00	1.00	1.00	1.00	1.00	2.60	2.76	2.60	2.76	2.76
Full fat soya	2.03	2.50	2.03	2.62	2.62	3.00	3.30	3.00	3.30	3.30	6.00	5.70	6.00	5.70	5.70
Palm oil	2.57	2.38	2.57	2.38	2.38	2.80	2.62	2.80	2.62	2.62	3.60	3.40	3.60	3.40	3.40
Limestone	0.99	0.98	0.98	0.87	0.87	0.76	0.73	0.76	0.73	0.73	0.12	0.12	0.12	0.12	0.12
Salt	0.20	0.20	0.20	0.20	0.20	0.20	0.20	0.20	0.20	0.20	0.19	0.18	0.19	0.18	0.18
Sodium bicarb	0.20	0.20	0.20	0.20	0.20	0.20	0.20	0.20	0.20	0.20	0.19	0.18	0.19	0.18	0.18
Sodium hum	0.10	0.10	0.10	0.10	0.10	0.10	0.10	0.10	0.10	0.10	0.10	0.10	0.10	0.10	0.10
Premix	1.49	1.49	1.49	1.49	1.49	1.34	1.34	1.34	1.34	1.34	1.44	1.44	1.44	1.44	1.44
l-Lysine	0.34	0.35	0.35	0.37	0.37	0.29	0.31	0.29	0.31	0.31	0.29	0.33	0.29	0.33	0.33
dl-Methionine	0.35	0.34	0.34	0.34	0.34	0.29	0.29	0.29	0.29	0.29	0.25	0.25	0.25	0.25	0.25
l-Threonine	0.19	0.19	0.19	0.19	0.19	0.14	0.15	0.14	0.15	0.15	0.12	0.13	0.12	0.13	0.13
Sunpro	0.00	0.00	0.005	0.005	0.01	0.00	0.00	0.005	0.005	0.01	0.00	0.00	0.005	0.005	0.01
Total	100	100	100	100	100	100	100	100	100	100	100	100	100	100	100
Nutrients calculation															
ME, Kcal	3,045	3,045	3,045	3,045	3,045	3,110	3,110	3,110	3,110	3,110	3,200	3,200	3,200	3,200	3,200
Moisture, %	11.84	11.78	11.78	11.68	11.68	11.88	11.49	11.88	11.49	11.49	11.88	11.73	11.73	11.31	11.31
Crude protein, %	23.5	22.5	23.5	22.5	22.5	21.57	20.57	21.57	20.57	20.57	19.5	18.5	19.5	18.5	18.5
Crude fiber, %	2.5	2.64	2.57	2.64	2.64	2.48	2.79	2.48	2.79	2.79	2.45	2.87	2.45	2.87	2.87
Crude fat, %	5.27	5.14	5.27	5.14	5.14	5.95	5.71	5.95	5.71	5.71	7.38	7.49	7.38	7.49	7.49
Ash, %	5.78	5.73	5.73	5.67	5.67	5.19	5.1	5.1	4.98	4.98	4.52	4.29	4.52	4.29	4.29
Ca, %	0.96	0.96	0.96	0.95	0.95	0.87	0.87	0.87	0.87	0.87	0.78	0.79	0.79	0.79	0.79
Av.P, %	0.47	0.47	0.47	0.47	0.47	0.44	0.43	0.43	0.43	0.43	0.45	0.43	0.43	0.43	0.43
Dig. Lys, %	1.35	1.35	1.35	1.35	1.35	1.2	1.2	1.2	1.2	1.2	1.08	1.08	1.08	1.08	1.08
Dig. Met, %	0.72	0.72	0.72	0.73	0.73	0.65	0.65	0.65	0.65	0.67	0.62	0.62	0.62	0.63	0.63

### Sample Collection and Measurements

#### Growth performance

The consumed feed was recorded daily and BW was monitored at 7-d intervals, then the collected data were used for the calculation of body weight (**BW**) gain and feed conversion ratio, and they were analyzed on days 21 and 35.

#### Blood biochemicals

Blood samples were collected on days 21 and 35 with 5 mL from the branchial vein of 35 broilers (one bird per replicate) using a sterile syringe and stored in an ethylenediaminetetraacetic acid (EDTA) tube to prevent bood clots. The sample tubes were taken to the laboratory and centrifuged at 2,000 × *g* for 10 min at 4 °C to obtain the supernatant plasma. Then, the collected plasma samples were transferred to an Eppendorf tube and stored at −20 °C before analysis. Plasma metabolites were analyzed by DiaSys Diagnostic System, Holzheim, Germany, using a commercial kit: glucose, total protein, albumin, cholesterol, uric acid, urea, phosphate, phosphorus, calcium, and creatinine.

#### Carcass yield and abdominal fat

At the end of the experiment on day 35, 70 birds (two birds per replicate) with BW close to the median BW were slaughtered to measure carcass yield. Carcass yield was calculated by dividing carcass weight (after removal of feathers, viscera, organs, and feet) by live weight (g/kg). At the same time, abdominal fat content around the gizzard and the fat layers between the abdominal muscles and intestines were collected and weighed. Abdominal fat yield was calculated by dividing abdominal fat weight by live weight (g/kg).

#### Intestinal morphology and organ weight

On days 21 and 35, 35 broilers (one bird per replicate) with BW close to the median BW were euthanized by cervical dislocation and the digestive tract was removed from the carcass. The intestinal morphology measurements were carried out from jejunum as the main part of intestine for nutrients absorption with a more obvious growth of intestinal morphology. The middle part of the jejunum was cut about 2 cm and placed in the tube which contains 10% buffered formalin solutions. Histological experiment samples were performed on 5 µm sections, stained by hematoxylin and eosin. Villi length, villi width, crypt depth, and the ratio of villus width length to crypt depth were determined using a microscope. The criteria and procedure to measure organ weight were determined according to Wilson et al. (2018) explained that the liver, pancreas, spleen, thymus, small intestine, and large intestine were dissected and measured for weight. The organ weight was divided by live weight (g/kg).

#### Nutrient digestibility

Thirty-five broilers (one bird per replicate) were transferred to metabolic cages. After the feeding trial for 3 d, total excreta from each cage were collected for 72 h on days 21 and 35. The collected excreta was dried in a dry oven at 55 °C for 3 d and ground to pass through a 0.5-mm sieve. The diet and excreta samples were analyzed for dry matter (**DM**), CP (AOAC, 2005), and gross energy using a Bomb Calorimeter (IKA Werke, Parr Instruments, Germany). The digestibility of DM, AME (Tevernari et al., 2018), and CP (Brink et al., 2022) was determined using the following formula:


$$\begin{array}{l} DM\left(\%\right)\;=\frac{\left(Feed\;intake\times DM\;diet\right)-\left(Excreta\;output\times DM\;excreta\right)}{Feed\;intake}\times \;100 \end{array}$$

$$\begin{array}{l} AME\left(\%\right)=\frac{\left(Feed\;intake\times GE\;diet\right)-(Excreta\;output\times GE\;excreta)}{Feed\;intake}\times 100 \end{array}$$

$$\begin{array}{l} CP\left(\%\right)=\frac{\left(Feed\;intake\times N\;diet\right)-\left(Exreta\;output\times N\;excreta\right)}{Feed\;intake\times N\;diet}\times 100 \end{array}$$

### Statistical Analysis

The obtained data would be analyzed using one-way analysis of variance. Duncan’s new Multiple Range Test would be subsequently used to separate mean with significant differences. An α level of *P* ≤ 0.05 was used as the criterion for statistical significance.

## Results

### Growth Performance

The effect of reduced-protein diets with/without protease on the growth performance of broiler was analyzed from 1 to 35 d and the results are shown in Table 2. There was no significant difference in body weight gain (**BWG**), feed intake (**FI**), and feed conversion rate (**FCR**) in broiler fed different diets (*P* > 0.05).

**Table 2.** Effect of reduced-protein with protease supplementation on growth performance of broiler

Item	PC	PC + 50	NC	NC + 50	NC + 100	*P*-value
IBW, g	46.30 ± 0.59	46.50 ± 0.56	46.76 ± 0.43	46.49 ± 0.44	46.45 ± 0.37	0.66
Days 1 to 21						
BWG, g	980.36 ± 35.16	999.21 ± 20.22	955.81 ± 42.26	946.87 ± 31.86	948.19 ± 23.14	0.76
FI, g	1,254.33 ± 56.63	1,276.13 ± 49.70	1,246.03 ± 48.98	1,246.33 ± 27.70	1,261.93 ± 19.57	0.25
FCR	1.28 ± 0.06	1.27 ± 0.05	1.30 ± 0.07	1.32 ± 0.04	1.33 ± 0.05	0.17
Days 22 to 35						
BWG, g	1,301.13 ± 41.99	1,328.65 ± 130.91	1,249.41 ± 121.56	1,336.05 ± 215.49	1,322.24 ± 74.12	0.79
FI, g	2,458.34 ± 111.63	2,500.06 ± 60.84	2,509.27 ± 50.92	2,516.16 ± 67.51	2,542.37 ± 76.93	0.40
FCR	1.89 ± 0.11	1.90 ± 0.20	2.02 ± 0.18	1.93 ± 0.31	1.93 ± 0.08	0.75
Days 1 to 35						
BWG, g	2,281.48 ± 39.41	2,327.86 ± 132.6	2,205.22 ± 146.67	2,282.93 ± 205.68	2,270.43 ± 67.99	0.59
FI, g	3,712.64 ± 123.81	3,755.25 ± 76.75	3,776.16 ± 40.83	3,762.42 ± 82.57	3,804.29 ± 84.19	0.44
FCR	1.63 ± 0.08	1.63 ± 0.09	1.71 ± 0.11	1.66 ± 0.14	1.68 ± 0.05	0.56

IBW: initial body weight; BWG: body weight gain; ADG: average daily gain; ADFI: average daily feed intake; FCR: feed conversion rate; PC: positive control (corn–soybean meal-based diet); PC + 50: PC + 50 g/t protease; NC: negative control (PC with reduction of 1% protein); NC + 50: NC + 50 g/t protease; NC + 100: NC + 100 g/t protease.

### Carcass Yield

The carcass yield and abdominal fat are shown in Table 3. Diet treatments did not affect carcass yield (*P* > 0.05). The PC + 50 group had significantly lower abdominal fat than the NC, NC + 50 and NC + 100 group (*P* < 0.05), while there was no significant difference between PC group and PC + 50 group on carcass yield (*P* > 0.05).

**Table 3.** Effect of reduced-protein with protease supplementation on carcass yield and abdominal fat of broiler

Item	PC	PC + 50	NC	NC + 50	NC + 100	*P*-value
Carcass, g/kg	713.35 ± 23.56	718.98 ± 13.11	704.47 ± 28.54	719.13 ± 10.50	717.74 ± 35.58	0.76
Abdominal fat, g/kg	5.26 ± 1.07^ab^	4.98 ± 0.98^a^	7.26 ± 1.09^c^	6.91 ± 0.71^bc^	7.44 ± 2.54^c^	0,02

PC: positive control (corn–soybean meal-based diet); PC + 50: PC + 50 g/t protease; NC: negative control (PC with reduction of 1% protein); NC + 50: NC + 50 g/t protease; NC + 100: NC + 100 g/t protease.

### Dietary Nutrients Digestibility

The nutrient digestibility of broiler fed reduced-protein diets with/without protease is shown in Table 4. There were no significant differences in digestibility of DM, CP, and energy in broiler fed different diets, no matter on 21 and 35 d (*P* > 0.05).

**Table 4.** Effect of reduced-protein with protease supplementation on dietary nutrient digestibility of broiler on days 21 and 35

Days	Nutrients	PC	PC + 50	NC	NC + 50	NC + 100	*P*-value
21	Dry matter, %	74.66 ± 1.79	74.20 ± 2.41	74.60 ± 1.11	74.91 ± 22.17	75.79 ± 2.14	0.68
	Crude protein, %	74.75 ± 2.67	73.14 ± 2.22	71.53 ± 0.61	72.31 ± 2.63	74.73 ± 4.97	0.34
	Energy, %	77.04 ± 1.68	76.42 ± 1.70	76.76 ± 1.09	76.72 ± 3.16	77.87 ± 2.10	0.75
35	Dry matter, %	81.13 ± 6.03	81.77 ± 3.75	78.03 ± 2.19	80.44 ± 2.85	80.01 ± 3.68	0.52
	Crude protein, %	74.15 ± 8.11	76.00 ± 4.16	70.67 ± 3.11	74.30 ± 4.69	73.36 ± 5.23	0.52
	Energy, %	82.93 ± 5.74	83.00 ± 3.97	79.36 ± 1.99	81.77 ± 2.55	81.93 ± 3.20	0.44

PC: positive control (corn-soybean meal-based diet); PC + 50: PC + 50 g/t protease; NC: negative control (PC with reduction of 1% protein); NC + 50: NC + 50 g/t protease; NC + 100: NC + 100 g/t protease.

### Organ Weights

The effects of reduced-protein with/without protease on broiler organ development on 21 and 35 d were shown in Table 5. On 21 d, dietary treatments did not significantly affect liver weight (*P* > 0.05), whereas they significantly affected the pancreas, spleen, thymus, small intestine, and large intestine (*P* < 0.05). Broiler pancreas was higher when fed PC and NC++50 diet than fed PC + 50 and NC diet (*P* < 0.05). NC group had significantly higher spleen than NC + 100 group, while PC, PC + 50, NC + 100 group did not have a significant difference with NC group or NC + 100 group. Thymus was significantly higher in broiler fed PC, NC, NC + 100 diet than fed NC + 50 diet (*P* < 0.05). Small intestine was significantly higher in NC + 50 and NC + 100 group than PC group (*P* < 0.05). NC + 50 group had a significantly higher large intestine than NC group, while they did not have a significant difference with PC, PC + 50, and NC + 100 group (*P* > 0.05). On 35 d, only spleen showed a significant difference among treatments, which was significantly higher in broiler fed PC and NC diet than fed PC + 50 diet (*P* < 0.05), but it was not significantly different in broiler fed NC + 50 and NC + 100 diet (*P* > 0.05).

**Table 5.** Effect of reduced-protein with protease supplementation on organ weight in broiler on days 21 and 35

Days	Organ	PC	PC + 50	NC	NC + 50	NC + 100	P-value
21	Liver, g/kg	23.4 ± 1.35	24.02 ± 1.56	24.64 ± 1.78	25.54 ± 1.57	25.61 ± 2.87	0.16
	Pancreas, g/kg	3.22 ± 0.72^b^	2.50 ± 0.20^a^	2.58 ± 0.29^a^	3.16 ± 0.44^b^	2.68 ± 0.53^ab^	0.02
	Spleen, g/kg	0.95 ± 0.18^ab^	0.87 ± 0.15^ab^	1.21 ± 0.40^b^	1.06 ± 0.23^ab^	0.74 ± 0.21^a^	0.02
	Thymus, g/kg	1.73 ± 1.16^a^	2.92 ± 0.74^ab^	1.72 ± 0.81^a^	3.88 ± 1.39^b^	1.81 ± 0.88^a^	0.00
	Small intestine, g/kg	27.96 ± 1.93^a^	29.01 ± 1.96^ab^	28.83 ± 1.67^ab^	31.75 ± 1.70^b^	31.51 ± 3.64^b^	0.01
	Large intestine, g/kg	1.12 ± 0.27^ab^	1.10 ± 0.31^ab^	1.09 ± 0.19^a^	1.68 ± 0.65^b^	1.17 ± 0.30^ab^	0.03
35	Liver, g/kg	27.41 ± 4.21	23.83 ± 3.48	26.47 ± 3.70	24.53 ± 2.22	25.99 ± 2.10	0.26
	Pancreas, g/kg	2.72 ± 0.30	2.16 ± 0.61	2.26 ± 0.33	2.20 ± 0.32	2.28 ± 0.41	0.10
	Spleen, g/kg	1.39 ± 0.23^b^	0.84 ± 0.39^a^	1.28 ± 0.31^b^	1.17 ± 0.20^ab^	1.12 ± 0.24^ab^	0.01
	Thymus, g/kg	2.25 ± 0.32	2.04 ± 0.51	3.04 ± 1.36	2.21 ± 0.56	2.17 ± 0.56	0.13
	Small intestine, g/kg	25.03 ± 3.43	21.36 ± 2.40	25.59 ± 3.07	23.74 ± 3.69	23.01 ± 1.43	0.08
	Large intestine, g/kg	1.27 ± 0.38	1.25 ± 0.51	1.50 ± 0.30	1.26 ± 0.27	1.26 ± 0.25	0.63

PC: positive control (corn–soybean meal-based diet); PC + 50: PC + 50 g/t protease; NC: negative control (PC with reduction of 1% protein); NC + 50: NC + 50 g/t protease; NC + 100: NC + 100 g/t protease.

### Intestine Morphology

The intestine morphology of broiler fed reduced-protein diets with/without protease is shown in Table 6. There was no significant difference in villus height (**VH**), crypt depth (**CD**), and villus height to crypt depth ratio (**VH:CD**) among groups on 21 d (*P* > 0.05). But VH and VH:CD had significant differences on 35 d, VH of PC + 100 was higher than NC and similar to PC and PC + 50. On the other hand, VH:CD in NC + 50 and NC + 100 were improved compared to NC but similar to PC (*P < 0.05*).

### Blood Biochemical

Plasma metabolites including glucose, albumin, cholesterol, urea, uric acid, phosphate, phosphorus, calcium, creatinine, and total protein on 35 d of broiler fed reduced-protein diets with/without protease is shown in Table 7. There was no significant difference of most metabolites, except for urea and uric acid. Urea was significantly higher in PC group than in NC group (*P* < 0.05), and its level was in the middle and not significantly different in PC + 50, NC + 50, NC + 100 group (*P* > 0.05). Broiler fed NC and NC + 50 group had significantly lower uric acid than the broiler fed PC and PC + 50 group (*P* < 0.05).

## Discussion

Enzyme supplementation has been acknowledged nutritionally, ecologically, and commercially. Young birds require more digestible protein to grow properly. The current study documented those birds fed by protease supplementation on a reduced-protein diet did not significantly affect growth performance. Previous authors have reported no significant interaction between the different feeding regimes and protease on birds’ growth performance (Amer et al., 2021). In contrast to this analysis, adding protease to broiler diets contributed to improving feed efficiency and nutrient digestibility, and the diets with 150 g/t of proteases had higher growth performances (Cho et al., 2021). Some studies have reported that low CP diets with exogenous proteases increase weight growth and FCR when compared to standard protein diets (Fru-nji et al., 2011).

The cumulative supplementation of multi-protease enzymes (alkaline and neutral form) contributed to a significant increase of BW through enhancing CP and energy digestibility, reflecting by 300 g/t of multi-protease supplementation showed better growth performance and nutrients digestibility than 150 g/t in protein-deficient feed (Cho et al., 2021). Furthermore, FCR was lower in broilers fed lower levels of protein, which might be related to lower feed intake and decreased weight growth (Jabbar et al., 2021b). Furthermore, many previous studies reported the reduced-protein level could be more than 3% without side effects on growth in terms of the amino acids requirement was fulfilled (Attia et al., 2020; Woyengo et al., 2023), suggesting more possibilities in low-protein diet formulation and protease application in the future. In the present study, growth performance in birds fed NC was similar to PC, protease supplementation had no effect on growth performance.

Based on prior study, there was no significant effect on carcass yield for protease in diets formulated with varying nutritional requirements for broilers. CP levels have a linear influence on abdominal fat (Dessimoni et al., 2019). Similar to our study that reducing protein level improved abdominal fat, supplementing protease at 100 g/t in negative control had significantly higher abdominal fat than negative control and even higher than positive control. It appeared that excess energy besides protein deposition was stored as abdominal fat (Jabbar et al., 2021b), although higher abdominal fat is not a desirable trait in broilers as consumers perceive fatty chicken as unhealthy. The previous research informed that CP digestibility was significantly increased in birds supplemented with protease (Jabbar et al., 2021b), and CP digestibility was also significantly influenced by the levels of additional protease (Ding et al., 2016). The value of CP digestibility was relatively higher in protease addition group, which was 2% higher than positive control group and more than 5% higher than negative control group. Furthermore, the addition of protease based on the negative control increased the digestibility of CP, with values higher than the negative control group and comparable to the positive control. However, we did not find the difference in nutrient digestibility when increasing protease dosage from 50 to 100 g/t.

Earlier research on protease supplementation under amino acids reduction in diets formulated with varying nutritional requirements for broilers found no significant effect on pancreatic weight (Dessimoni et al., 2019). Both spleen and liver weight did not show significant interaction between CP levels and exogenous enzyme supplementation on the diet, on 21 and 35 d of age (Ndazigaruye et al., 2019). The previous research also found no significant impact on spleen and thymus weight by protease supplementation (Dinani et al., 2019). In the present study, there were no difference on liver weight, pancreas weight, thymus weight, small intestine, and large intestine weight irrespective of positive control, protein-deficient groups, or protease supplementation at 35-d. The increased size and weight of the spleen may be attributable to the pathogenic bacteria infection, which caused the spleen to work harder, resulting in the increased size and weight (Perdinan et al., 2019). Only spleen weight showed differences caused by treatments that PC + 50 group showed lower spleen weight than PC group and NC group. Ndazigaruye et al. (2019) thought the effects on immune organ weight were either positive or negative depending on the level of CP. The reasons for the difference of spleen weight depending on the CP level are not yet well understood in the present study and require further research.

According to the earlier studies that the effects of low-protein diets and protease supplementation on broiler chickens in a hot and humid tropical environment had significant impact on jejunal VH, but did not significantly affect CD and VH:CD (Law et al., 2018). As we know that absorption of dietary nutrients is directly related to VH (Choct, 2006), in broiler chickens, a reduction of intestinal VH and VH:CD causes poor nutrients absorption and as a result, poor growth performance (Xu et al., 2017). VH, CD, and VH:CD are significant indices for assessing small intestine function, therefore, higher VH and VH:CD and lower CD appear to suggest a superior intestinal structure and more nutritional digestion and absorption ability (Viveros et al., 2011). On 35 d, dietary protein was reduced by 1% in NC + 100 group. VH was similar to the positive control groups, but in NC and NC + 50 had lower VH than the PC groups. The highest VH:CD was observed in PC + 50. NC + 50 and NC + 100 had similar VH:CD to PC. The above evaluation of protein digestibility was also in accordance with the current observation of gut morphology that PC + 50 group had higher VH than the others except NC + 100 and VH:CD compared to others with relative higher protein digestibility.

The previous studies found no significant effect of albumin on the interaction of protease enzymes with low-protein diets (Amer et al., 2021). Blood serum triglycerides, albumin, total cholesterol, high-density lipoprotein, and globulin levels in the present study were not affected by diet supplemented with/without exogenous protease enzyme or the changes in dietary protein content. Previous study had documented that substantial reduction in uric acid in broiler chicks fed reduced CP diets. Furthermore, protease supplementation increased uric acid, especially when added to the low-protein diet. Serum uric acid values are frequently reported to be an indicator of amino acid absorption in broilers given amino acid-sufficient and amino acid-deficient diets (Donsbough et al., 2010). The present blood urea and uric acid were consistent with former studies that they were lower in low-protein diet, but supplementing protease increased their concentration, especially for higher protease dosage as 100 g/t, reinforcing the benefits of protease on protein utilization as reflected by the metabolite levels.

## Conclusions

Exogenous protease supplementation could reduce 1% protein in broiler’s diet without significantly negative effects on growth, nutrient digestibility, carcass yield, organ development, and blood metabolites. Protease supplementation benefited for intestinal growth by promoting VH and VH:CD ratio. The effective dosage of the present commercial protease was 50 g/t for broiler feed.

**Table 6.** Effect of reduced-protein with protease supplementation on jejunal intestinal morphology in broiler on days 21 and 35

Days	Morphology	PC	PC + 50	NC	NC + 50	NC + 100	P-value
21	VH	1,192.94 ± 159.5	1,288.84 ± 207.8	1,117.82 ± 148.5	1,191.15 ± 92.6	1,147.95 ± 51.0	0.25
	CD	177.77 ± 16.85	185.72 ± 12.76	189.12 ± 12.39	193.64 ± 13.08	181.34 ± 28.05	0.49
	VH:CD	6.74 ± 0.96	6.98 ± 1.31	5.90 ± 0.63	6.19 ± 0.79	6.44 ± 0.87	0.24
35	VH	1,341.13 ± 187.79^b^	1,368.53 ± 155.68^b^	1,118.28 ± 50.48^a^	1,284.26 ± 86.07^ab^	1,395.47 ± 148.35^b^	0.00
	CD	211.69 ± 28.31	193.72 ± 14.72	220.97 ± 26.26	198.65 ± 25.10	219.40 ± 33.56	0.22
	VH:CD	6.41 ± 1.14^ab^	7.11 ± 1.02^b^	5.15 ± 0.90^a^	6.56 ± 0.99^ab^	6.45 ± 0.87^ab^	0.01

VH: villus height; CD: crypt depth; VH:CD: villus height to crypt depth ratio; PC: positive control (corn–soybean meal-based diet); PC + 50: PC + 50 g/t protease; NC: negative control (PC with reduction of 1% protein); NC + 50: NC + 50 g/t protease; NC + 100: NC + 100 g/t protease.

**Table 7.** Effect of reduced-protein with protease supplementation on blood biochemical in broiler on day 35

Plasma metabolites	PC	PC + 50	NC	NC + 50	NC + 100	*P*-value
Glucose, mg/dL	273.20 ± 36.02	246.61 ± 31.63	268.48 ± 27.07	262.89 ± 22.87	259.44 ± 13.18	0.44
Albumin, g/dL	1.22 ± 0.09	1.24 ± 0.09	1.22 ± 0.12	1.21 ± 0.09	1.32 ± 0.11	0.22
Cholesterol, mg/dL	130.47 ± 17.24	128.29 ± 8.37	127.68 ± 7.01	133.57 ± 14.75	133.48 ± 11.76	0.84
Urea, mg/dL	1.96 ± 0.42^b^	1.82 ± 0.20^ab^	1.46 ± 0.18^a^	1.66 ± 0.41^ab^	1.71 ± 0.14^ab^	0.05
Uric acid, mg/dL	6.09 ± 0.45^b^	5.90 ± 0.66^b^	4.51 ± 0.62^a^	5.03 ± 0.44^a^	5.27 ± 0.46^ab^	0.00
Phosphate, mg/dL	8.97 ± 1.39	7.95 ± 1.95	8.75 ± 1.54	8.31 ± 1.49	8.44 ± 1.45	0.77
Phosphorus, mg/dL	4.19 ± 0.65	3.71 ± 0.91	4.09 ± 0.72	3.88 ± 0.70	3.95 ± 0.68	0.78
Calcium, mg/dL	12.57 ± 1.78	12.28 ± 1.60	11.56 ± 2.87	13.01 ± 1.61	13.36 ± 2.08	0.54
Creatinine, mg/dL	0.16 ± 0.03	0.15 ± 0.03	0.14 ± 0.03	0.15 ± 0.02	0.16 ± 0.04	0.42
Total protein, g/dL	3.17 ± 0.51	3.23 ± 0.56	2.92 ± 0.57	3.35 ± 0.50	3.72 ± 0.58	0.12

PC: positive control (corn–soybean meal-based diet); PC + 50: PC + 50 g/t protease; NC: negative control (PC with reduction of 1% protein); NC + 50: NC + 50 g/t protease; NC + 100: NC + 100 g/t protease.
